# *Notes from the Field*: Surveillance of Silicosis Using Electronic Case Reporting — California, December 2022–July 2023

**DOI:** 10.15585/mmwr.mm7246a4

**Published:** 2023-11-17

**Authors:** Jennifer Flattery, Chelsea Woolsey, Melanie Epstein-Corbin, David J. Blackley, Robert J. Harrison, Kristin J. Cummings

**Affiliations:** ^1^Occupational Health Branch, California Department of Public Health, Richmond, California; ^2^Public Health Institute, Oakland, California; ^3^Center for Infectious Diseases, California Department of Public Health; ^4^National Institute for Occupational Safety and Health, CDC.

Electronic case reporting (eCR) (*1*) is a promising rapid reporting mechanism, whereby electronic health records (EHRs) automatically generate and transmit a disease report to jurisdictional public health agencies in real time using previously established criteria. All 50 U.S. states and other jurisdictions are connected to the eCR infrastructure. The Reportable Conditions Knowledge Management System (RCKMS),* a component of the eCR infrastructure, is a real-time decision support service that processes reports according to jurisdictional reporting requirements with criteria defined by Council of State and Territorial Epidemiologists’ position statements (*1*). Health care organizations automatically generate and send an initial case report to the eCR infrastructure when trigger criteria, such as diagnosis codes or laboratory results, are met within their EHRs. Therefore, for all participating California health care organizations, if a health care encounter involves COVID-19 or mpox, an initial case report is generated and sent to the eCR infrastructure for processing. When there is a match between the initial case report triggered by an EHR, and a reportable condition rule is entered into RCKMS by a jurisdictional public health agency, the initial case report is routed by the eCR infrastructure to the public health agency. Other conditions can be added to public health agency reporting rules.

Silicosis is a progressive, incurable, fibrotic lung disease caused by inhalation of respirable crystalline silica dust produced in industries such as construction, quarrying, and coal mining (*2*). A resurgence of silicosis among young workers fabricating engineered stone (quartz) countertops in California and in countries including Australia, Israel, and Spain has focused attention on the need for timely case identification for primary and secondary prevention (*2*–*5*). In December 2022, the California Department of Public Health (CDPH) added reporting rules for silicosis to RCKMS, so that any initial case report received by the eCR infrastructure from health care provider EHRs that includes a silicosis diagnosis in the patient’s problem list is sent to CDPH for silicosis surveillance. The purpose of this study was to evaluate the utility of eCR for identifying cases of silicosis in California. This study was reviewed and approved by the California Committee for the Protection of Human Subjects institutional review board.^†^

## Investigation and Outcomes

During October 2022–July 2023, CDPH received electronic initial case reports including silicosis for 41 persons. Medical records were reviewed to confirm cases, collect employment and exposure information, and initiate public health follow-up. Overall, nine (22%) of the 41 patients reported were also identified through other reporting sources, including hospital discharge data and direct referral. To date, 35 (85%) silicosis cases were identified, including 19 (46%) confirmed^§^ and 16 (39%) clinically compatible (probable) cases that lack confirmatory information (such as occupation, imaging, or biopsy) in the medical record. Six (15%) of the 41 reports were considered unlikely cases after medical record review. The median age of the patients with confirmed or probable silicosis was 65 years (range = 33–89 years), and 32 (91%) were male. At least seven of the 19 confirmed silicosis cases were associated with fabrication of engineered stone (quartz) countertops, although occupational or exposure information was missing for two patients. Among the seven persons who were engineered stone workers, the median age was 44 years (range = 33–51 years), and all were Hispanic or Latino; one patient died, two underwent bilateral lung transplantation, and one was evaluated for a lung transplant, all because of their silicosis diagnoses.

## Preliminary Conclusions and Actions

The 41 persons reported to date largely represent COVID-19 initial case reports that also include silicosis in the patient’s problem list. RCKMS at one health care organization in California has triggered conditions beyond COVID-19 and mpox, including silicosis, which resulted in six more patients (15%) being reported. The number of silicosis cases identified is a fraction of the reports anticipated when more health care organizations implement silicosis trigger criteria in addition to COVID-19 and mpox trigger criteria. These preliminary results illustrate the utility of eCR for identifying silicosis cases, because 32 (78%) of the 41 persons reported through eCR were not identified through other reporting mechanisms. It is important that health care providers routinely ask patients about their work as an important determinant of health. Being aware of the risks associated with work exposures, as well as the regulations, medical monitoring, and prevention strategies that address those risks can help guide patient care. In addition, many public health jurisdictions throughout the United States can add reporting rules for silicosis in RCKMS to receive silicosis electronic initial case reports. Further surveillance and follow-up should be completed to evaluate the effect of earlier reporting on disease outcome and prevention. eCR might help to further elucidate the scope and breadth of this important public health condition among vulnerable workers, with the goal of developing and implementing effective prevention strategies. Moreover, public health jurisdictions can implement eCR criteria for other important public health conditions in addition to silicosis.

## Footnotes

*
https://www.rckms.org/about-rckms/

† 45 C.F.R. part 46.114; 21 C.F.R. part 56.114.

§ Silicosis surveillance case definition is a history of occupational exposure to airborne silica dust and either or both of the following criteria: 1) chest radiograph or other imaging technique interpreted as consistent with silicosis, and 2) pathologic findings characteristic of silicosis. https://www.cdc.gov/niosh/topics/surveillance/ords/statesurveillance/reportingguidelines-silicosis.html

## References

[1] CDC. Why is eCR important? Atlanta, GA: US Department of Health and Human Services, CDC; 2022. https://www.cdc.gov/ecr/why-is-ecr-important.html

[2] Leso V, Fontana L, Romano R, Gervetti P, Iavicoli I. Artificial stone associated silicosis: a systematic review. Int J Environ Res Public Health 2019;16:568–85. 10.3390/ijerph16040568 30781462 PMC6406954

[3] Fazio JC, Gandhi SA, Flattery J, Silicosis among immigrant engineered stone (quartz) countertop fabrication workers in California. JAMA Intern Med 2023;183:991–8. 10.1001/jamainternmed.2023.3295 37486642 PMC10366949

[4] Hua JT, Zell-Baran L, Go LHT, Demographic, exposure and clinical characteristics in a multinational registry of engineered stone workers with silicosis. Occup Environ Med 2022;79:586–93. 10.1136/oemed-2021-108190 35504722 PMC9453561

[5] Hoy RF, Dimitriadis C, Abramson M, Prevalence and risk factors for silicosis among a large cohort of stone benchtop industry workers. Occup Environ Med 2023;80:439–46. 10.1136/oemed-2023-108892 37328266 PMC10423513

